# Travel-associated Illness Trends and Clusters, 2000–2010

**DOI:** 10.3201/eid1907.121573

**Published:** 2013-07

**Authors:** Karin Leder, Joseph Torresi, John S. Brownstein, Mary E. Wilson, Jay S. Keystone, Elizabeth Barnett, Eli Schwartz, Patricia Schlagenhauf, Annelies Wilder-Smith, Francesco Castelli, Frank von Sonnenburg, David O. Freedman, Allen C. Cheng, for the GeoSentinel Surveillance Network

**Affiliations:** Royal Melbourne Hospital, Parkville, Victoria, Australia (K. Leder);; Monash University, Melbourne, Victoria, Australia (K. Leder, A.C. Cheng);; Austin Hospital, Heidelberg, Victoria, Australia (J. Torresi);; University of Melbourne, Parkville (J. Torresi);; Children’s Hospital, Boston, Massachusetts, USA (J.S. Brownstein);; Harvard Medical School, Boston (J.S. Brownstein);; Harvard School of Public Health, Boston (M.E. Wilson);; Toronto General Hospital, Toronto, Ontario, Canada (J.S. Keystone);; University of Toronto, Toronto (J.S. Keystone);; Boston Medical Center, Boston (E. Barnett);; Sheba Medical Center, Tel Hashomer, Isreal (E. Schwartz);; Tel Aviv University, Tel Aviv, Israel (E. Schwartz);; University of Zurich WHO Collaborating Centre for Travellers’ Health, Zurich, Switzerland (P. Schlagenhauf);; Lee Kong Chian School of Medicine, Singapore (A. Wilder-Smith);; University of Brescia, Brescia, Italy (F. Castelli);; University of Munich, Munich, Germany (F. von Sonnenburg);; University of Alabama at Birmingham, Birmingham, Alabama, USA (D.O. Freedman);; Alfred Hospital, Melbourne (A.C. Cheng)

**Keywords:** travel, trends, case clusters, illness, surveillance, VFRs, visiting friends and relatives

## Abstract

Longitudinal data examining travel-associated illness patterns are lacking. To address this need and determine trends and clusters in travel-related illness, we examined data for 2000–2010, prospectively collected for 42,223 ill travelers by 18 GeoSentinel sites. The most common destinations from which ill travelers returned were sub-Saharan Africa (26%), Southeast Asia (17%), south-central Asia (15%), and South America (10%). The proportion who traveled for tourism decreased significantly, and the proportion who traveled to visit friends and relatives increased. Among travelers returning from malaria-endemic regions, the proportionate morbidity (PM) for malaria decreased; in contrast, the PM trends for enteric fever and dengue (excluding a 2002 peak) increased. Case clustering was detected for malaria (Africa 2000, 2007), dengue (Thailand 2002, India 2003), and enteric fever (Nepal 2009). This multisite longitudinal analysis highlights the utility of sentinel surveillance of travelers for contributing information on disease activity trends and an evidence base for travel medicine recommendations.

International travel is markedly increasing. In 2010, an estimated 940 million tourists arrived at international destinations, more than twice the 435 million in 1990 (*1*). Trips to developing regions have risen from 31% of all travel in 1990 to 47% in 2010, and trips to the Asia–Pacific region, Africa, and the Middle East have doubled in the past decade (*1*). Reasons for travel have also changed; from 1990 to 2010, trips for tourism decreased from 56% to 51%, and trips by those with ties to the destination country (travel for the purpose of visiting friends and relatives) increased from 20% to 27% (*1**,**2*).

More than half of international travelers to developing countries become ill during their trip, and ≈8% seek medical care for a travel-associated illness either during or after travel (*3*). Changes in travelers’ illnesses over time would be expected to reflect changing patterns of global travel destinations, changes in local disease epidemiology in regions visited, and/or availability of preventive measures such as vaccination and chemoprophylaxis. To examine illness trends and clusters among travelers, we analyzed multisite longitudinal data collected by GeoSentinel sites during 2000–2010.

## Materials and Methods

**Figure 1:**
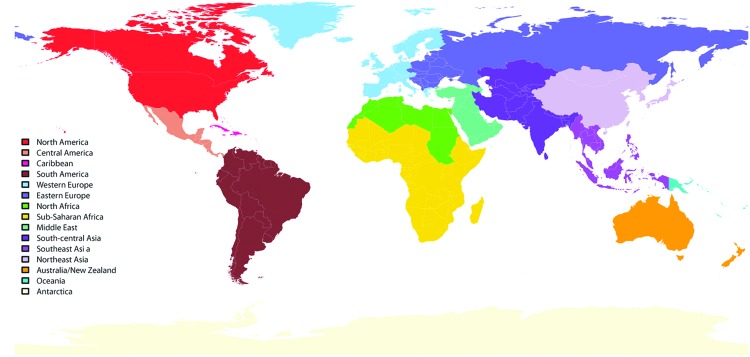
GeoSentinel regions.

The GeoSentinel Surveillance Network (www.geosentinel.org) is a global network of specialized travel and tropical medicine providers. It was established through the International Society of Travel Medicine and the US Centers for Disease Control and Prevention (CDC) (*4*). Since 1997, data have been collected on illnesses imported across international borders by travelers and immigrants. GeoSentinel sites have been added progressively over time; currently, 54 clinics in 26 countries contribute data (Figure 1). Information is recorded for demographics, travel history, reason for travel, clinical signs and symptoms, and diagnosis. All sites use the best available reference diagnostic tests and base the identification of country (or region) of illness acquisition on itinerary, known endemicity patterns, and incubation periods. GeoSentinel sites enter their de-identified questionnaire-based information into a central SQL (structured query language) database. The GeoSentinel data-collection protocol was reviewed by the institutional review board officer at the CDC and was classified as public health surveillance, not as human subjects research requiring submission to institutional review boards.

The mix of patients and diagnoses reported by individual GeoSentinel sites varies according to site location and clinic type (hospital or outpatient). To examine trends over time, we included only sites that consistently reported posttravel data throughout the 11-year period of interest. From these sites we examined inpatient and outpatient data for trends in demographics, reason for travel, and proportionate morbidity (PM) for certain key diagnoses. Specific infections were included in analyses on the basis of clinical relevance plus sufficient case numbers (Table 1). PM is expressed as number of cases/1,000 ill travelers returned from the region(s) of interest. Where model fit of the PM variation over time was adequate (assessed statistically as the proportion of variance explained by year and considered adequately fitted if the coefficient of determination [R2 statistic] was >50%), the rate of change in proportion was estimated by using linear regression with year as the independent variable and by using p value to assess the null hypothesis that there was no change over time. Statistical significance was set at p<0.05. Statistical procedures were performed by using Stata 10/IC for Windows (StataCorp LP, College Station, TX, USA).

**Table 1:** Major diagnoses for returning travelers visiting 18 GeoSentinel sites, 2000–2010*

Diagnosis	No. cases
Malaria	1,762
Giardiasis	1,296
Dengue fever	888
Campylobacteriosis	596
Cutaneous larva migrans	577
Rabies postexposure prophylaxis	349
Enteric fever†	262
Spotted fever rickettsiosis	220
Chikungunya	120
Acute hepatitis A	94
Confirmed influenza A/B	84

*Other diagnoses included nonspecific gastrointestinal or diarrheal syndromes (≈25% of all patients); nonspecific febrile illness or viral syndrome (≈10%); rash, itch, or skin infection (≈10%); respiratory syndrome (≈5%); and other infectious and noninfectious problems. †Salmonella enterica serovar Typhi, S. enterica ser. Paratyphi, or unspecified.

Case clustering was assessed by using the scan statistic on georeferenced data. Cases were georeferenced to the centroid of the likely country of acquisition, and rate estimates were based on the total number of ill returned travelers from that country who visited GeoSentinel sites. Diseases examined—dengue, malaria, and enteric fever—were chosen because of clinical importance and relative frequency. The scan statistic uses a Poisson model to estimate the number of cases of each disease relative to the population returning from a given area. To encompass changes in season, the temporal window was set to 3 months. The spatial window was set to a 1,000-km radius. The scan statistic was calculated by using SatScan 9.1.1 (Kulldorff M; Information Management Services Inc., Boston, MA, USA), and significance was assessed by using 1,000 Monte Carlo simulations.

## Results

Of 54 current GeoSentinel sites, 18 reported consistently during 2000–2010: 12 sites in North America, 2 in Australasia, and 4 in Europe/Middle East. A total of 42,223 ill returned travelers were reported and included in this study. The annual increase in patient numbers reported over the 11 years was statistically significant (+5%/year, p = 0.03).

### Reason for Travel

**Figure 2:**
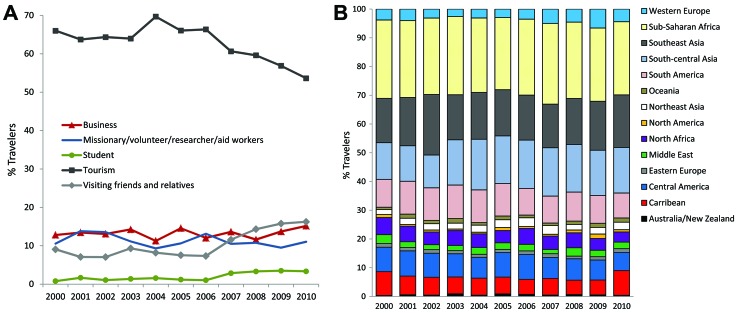
A) Reason for travel among 42,223 ill returned GeoSentinel patients, 2000–2010. Reason for travel missing for 188 (0.4%) patients. B) Destinations of travel among 42,223 ill returned GeoSentinel patients, 2000–2010. Region missing or unable to be determined (>1 region was visited) for 3,601 (8.5%) patients.

Tourists comprised 63% of ill returned travelers overall, but over the study period, this proportion decreased by 10% (p = 0.009). By contrast, the proportion of those who traveled to visit friends and relatives increased from 9.1% in 2000 to 16.3% in 2010 (p = 0.002). The proportion of ill returned business travelers and missionaries/volunteers remained unchanged (Figure 2, panel A); the proportion of patients who reported having received pretravel advice declined, but not significantly (–5%, p = 0.07).

### Destinations

The most common destinations from which ill travelers returned were sub-Saharan Africa (26%), Southeast Asia (17%), south-central Asia (15%), and South America (10%) (Figure 2, panel B). Over the 11-year period, the proportion of those returning from south-central Asia increased significantly (+5%, p = 0.028).

### Diseases

#### Malaria

**Figure 3:**
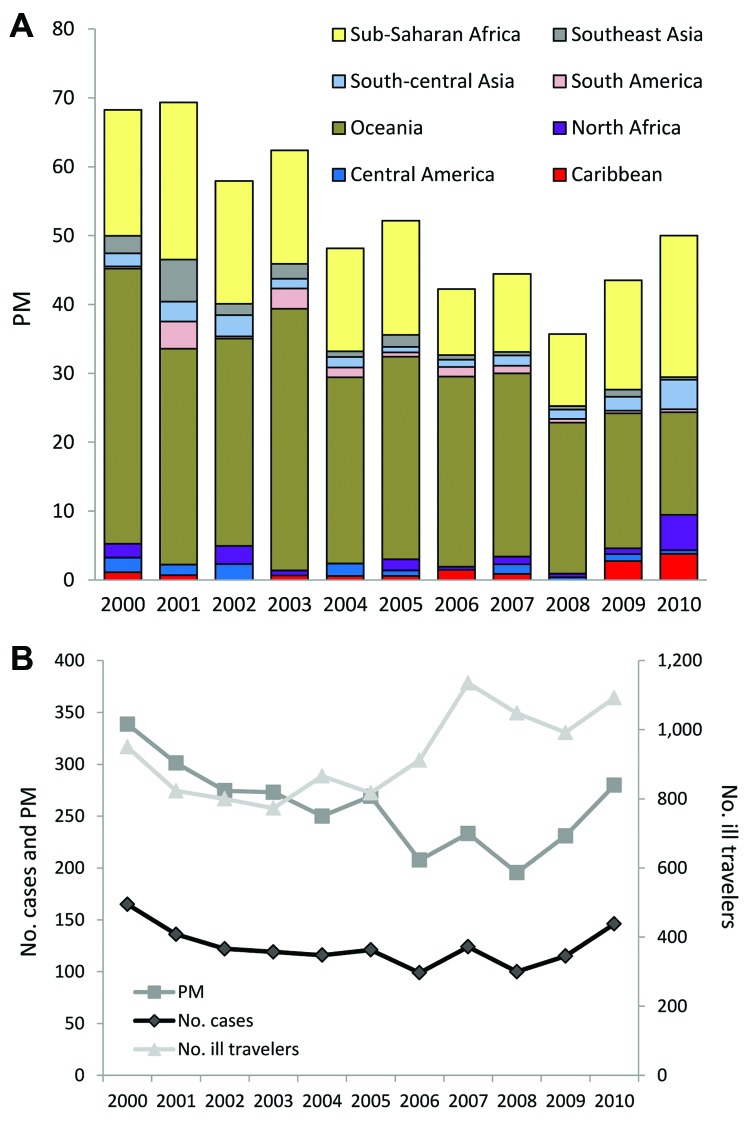
A) Proportionate morbidity (PM) for malaria (no. malaria cases/1,000 ill returned GeoSentinel patients) by region, 2000–2010. B) Absolute case numbers and proportionate morbidity for malaria (no. malaria cases/1,000 ill returned GeoSentinel patients) after travel to sub-Saharan Africa, 2000–2010. There were 1,363 total reported cases of malaria after travel to sub-Saharan Africa among the 18 GeoSentinel sites.

The most common sites for acquiring malaria were sub-Saharan Africa (77%), Oceania (6%), and south-central and Southeast Asia (5% each). However, the PM for malaria was greater for ill travelers returning from Oceania (average 248 malaria cases/1,000 ill travelers from the region) than for those returning from sub-Saharan Africa (average 135 cases/1,000 travelers). In 2000, the overall PM for malaria was 68 cases/1,000 ill travelers, and during 2000–2010, the rate decreased by an average of 30/1,000 (p = 0.002) (Figure 3, panel A). Despite overall increasing visits to GeoSentinel sites during the study period, absolute case numbers for malaria decreased (211 malaria cases reported in 2000; 151 in 2006, 124 in 2008, 189 in 2010) (Figure 3, panel B). The PM (and absolute case numbers) rose marginally during 2009–2010, compared with 2008, but did not negate the overall decreasing trend over the study period, which was most marked among ill travelers returning from Oceania (–204/1,000, p = 0.010), sub-Saharan Africa (–68/1,000, p = 0.003), and Southeast Asia (–31/1,000, p = 0.005).

PMs for malaria caused by *Plasmodium falciparum* and *P. vivax* decreased (–13/1,000, p = 0.012, and –13/1,000, p = 0.001, respectively). At a regional level, PMs for falciparum malaria decreased among ill travelers returning from sub-Saharan Africa (–39/1,000, p = 0.010), and PMs for vivax malaria decreased among those returning from Oceania (–169/1,000, p = 0.005), sub-Saharan Africa (–19/1,000, p < 0.001), and Southeast Asia (–24/1,000, p = 0.009). Decreasing PM trends for malaria were also small but significant among ill returning tourists (–37/1,000, p<0.001), travelers who visited friends and relatives (–103/1,000, p = 0.011), and business travelers (–43/1,000, p = 0.007).

#### Enteric Fever

**Figure 4:**
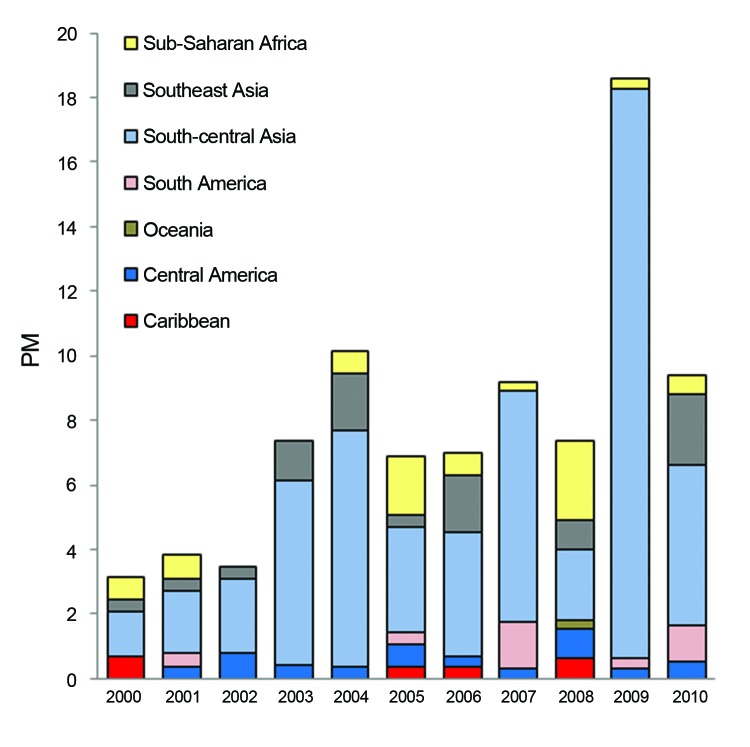
Proportionate morbidity (PM) for enteric fever (no. enteric fever cases/1,000 ill returned GeoSentinel patients) by region, 2000–2010.

For enteric fever (caused by *Salmonella enterica* serovar Typhi, S. enterica ser. Paratyphi, or unspecified), 67% of cases were imported from south-central Asia and 10% from each of Southeast Asia and sub-Saharan Africa (Figure 4). The PM for enteric fever increased over the 11 years (+10/1,000, p = 0.013). Exclusion of the 2009 cluster (Table 2) did not negate the overall significant trend. Regional trends could not be assessed because of considerable year-to-year variation in PM by region. Tourism accounted for 55% of cases and travel to visit friends and relatives for 27%.

**Table 2:** Main clusters detected among GeoSentinel patients, 2000–2010

Diagnosis, cluster	Location and dates of clusters	Cases/no. ill travelers (no. expected cases, p value)	Comments
Malaria			
Cluster A	Benin, Togo, Ghana, Burkina Faso, Nigeria, Cote d'Ivoire, 2007 mid Jul–mid Oct	44/185 (2.6, p<0.0001)	No definitive outbreaks discernible on ProMED-mail or in published literature corresponding to these clusters
Cluster B	Mauritania, Western Sahara, Mali, Senegal, 2000 mid Sep–mid Oct	19/53, (0.5, p<0.0001)	
Enteric fever	Nepal, 2009 Oct 5–20	24/40 (0.03, p<0.0001)	Associated with an outbreak of Salmonella paratyphi A among Israeli travelers to Nepal (*5*)
Dengue			
Cluster A	Thailand, 2002 Apr–Jul	44/257 (1.7, p<0.0001)	Reported in (*6*)
Cluster B	India, 2003 Sep–Nov	13/368 (0.9, p<0.0001)	

#### Dengue

**Figure 5:**
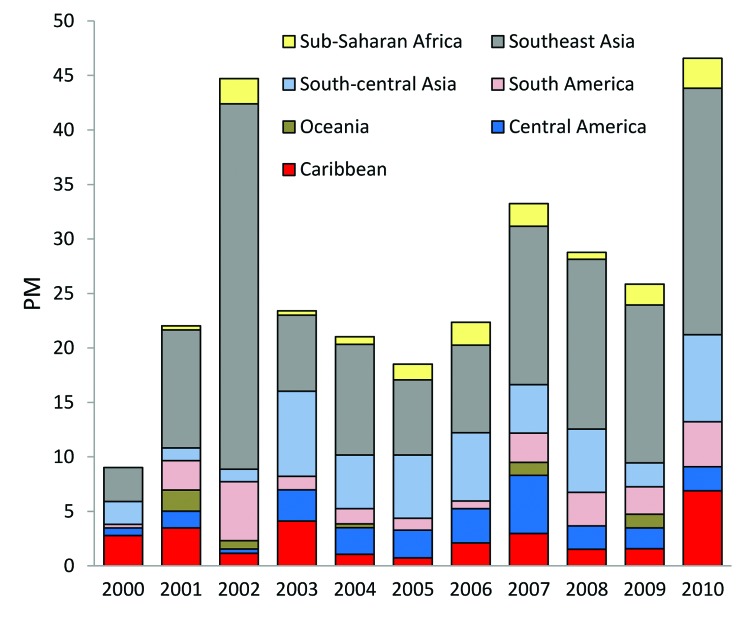
Proportionate morbidity (PM) for dengue (no. dengue cases/1,000 returned GeoSentinel patients) by region, 2000–2010.

With regard to dengue, 50% of patients had visited Southeast Asia; 17% south-central Asia; 9%–10% each Central America, South America, or the Caribbean; and 5% sub-Saharan Africa. There was considerable year-to-year PM variation not accounted for by a linear trend, largely because of a clear peak in 2002 (associated with an outbreak in Thailand) (*6*) (Figure 5). Excluding this peak, the underlying dengue PM (+26/1,000, p = 0.006) and case numbers (26 in 2000, 169 in 2010) increased significantly, especially among ill travelers returning from Southeast Asia (+71/1,000, p = 0.004) and sub-Saharan Africa (+8/1,000, p = 0.005).

#### Other

**Figure 6:**
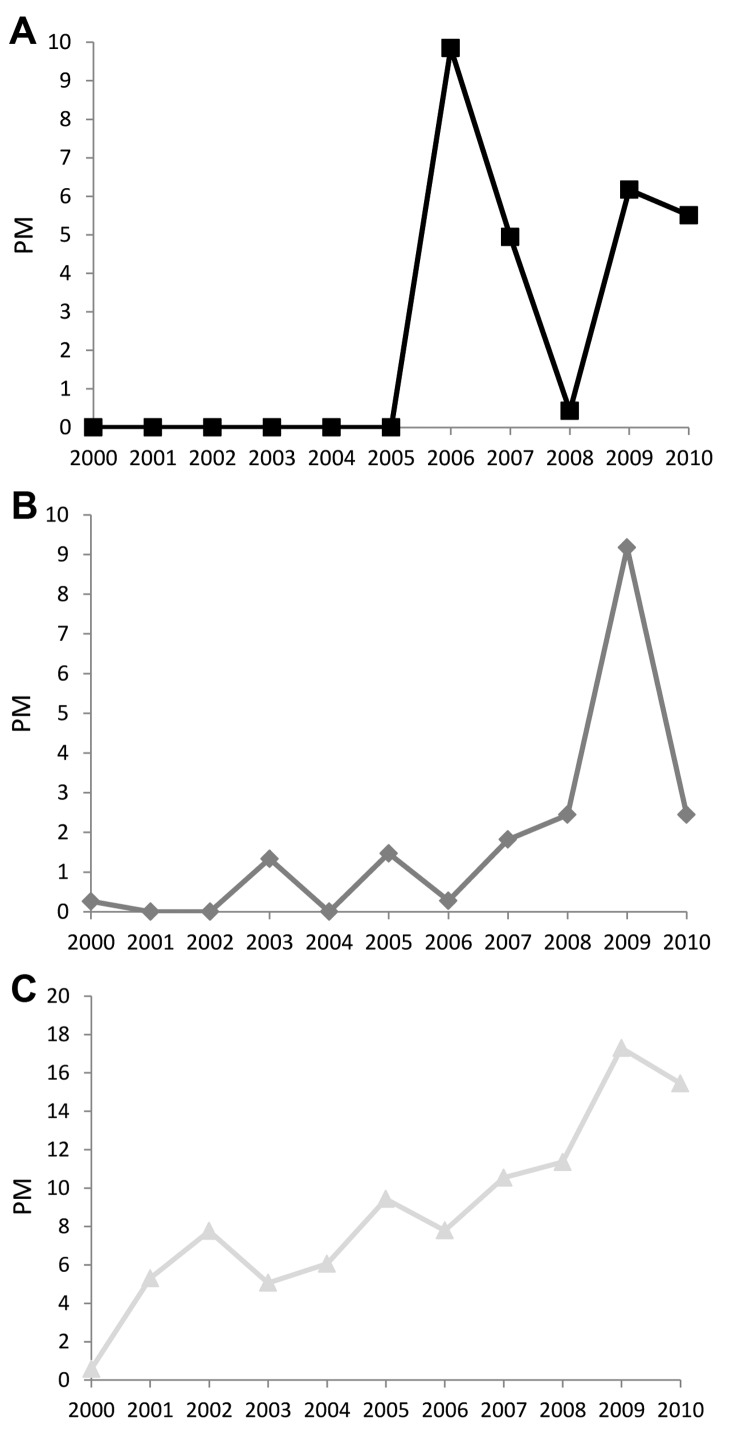
Proportionate morbidity (PM) (no. cases/1,000 returned GeoSentinel patients), 2000–2010. A) chikungunya, B) influenza, and C) rabies postexposure prophylaxis. Trends for chikungunya and influenza were not calculated because of substantial nonlinear year-to-year variation.

For chikungunya, the PM for total infections increased, as did the PM for cases acquired in Southeast Asia and south-central Asia. However, variation in PM was not well accounted for by a linear trend either overall or by region (Figure 6, panel A). 

The PM for confirmed influenza A or B also increased. Similar to chikungunya, the variation in PM was not well accounted for by a linear trend, mostly because of the high number of visits during the 2009 pandemic (Figure 6, panel B). 

 The PM for rabies postexposure prophylaxis (PEP) increased significantly (+153/1,000, p<0.001) (Figure 6, panel C), particularly among those returning from Southeast Asia (+49/1,000, p = 0.001). No significant trends were found for hepatitis A, campylobacteriosis, giardiasis, cutaneous larva migrans, or spotted fever rickettsiosis.

Cluster analyses were performed only for malaria, enteric fever, and dengue. Results are shown in Table 2.

## Discussion

Sentinel surveillance of travelers is increasingly being recognized as an integral element for identifying emerging infections and disease outbreaks (*7*). However, the historical absence of systematic longitudinal data on travelers means that there are no studies on long-term disease trends among travelers and no data on whether traveler importation of illness mirrors regional disease trends in local populations. GeoSentinel surveillance has been performed continuously for >10 years and enables examination of longitudinal disease trends and clusters among returning ill travelers.

This 11-year analysis of ≈42,000 returned ill travelers identified several significant findings. Almost 60% had visited sub-Saharan Africa, Southeast Asia, or south-central Asia, but the largest regional fluctuation was in south-central Asia (+5%, p = 0.028). The proportions of ill tourists (–10%) and ill travelers who had visited friends and relatives (+9%) changed inversely. Although those who visit friends and relatives are at high risk for many travel-related health problems, pretravel advice and adequate precautions are often lacking (*8**,**9*).

**Figure 7:**
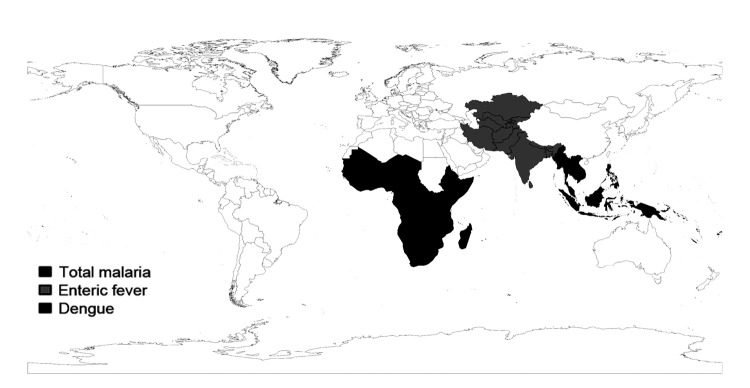
Regional results for malaria, enteric fever, and dengue. For malaria, the top region for acquisition was sub-Saharan Africa (77%), and the region with the top average proportionate morbidity (248/1,000 ill travelers) and the greatest trend (–39/1,000, p = 0.01) Oceania. For enteric fever, the top region for acquisition was south-central Asia (67%); regional trends were not assessed. For dengue, the top region for acquisition (50%) and the highest average proportionate morbidity and trend (+70.5/1,000, p = 0.004) was Southeast Asia.

Trends in morbidity rates for individual illnesses among travelers are influenced by many factors, including changes in disease incidence in regions visited, variations in uptake of preventive measures, and diagnostic factors. We report PM for specific illnesses, which is additionally influenced by changes in the number of travelers seen at GeoSentinel sites for other illnesses; therefore, interpretation of the longitudinal trends in PM requires caution. However, the proportion of the most common other illnesses seen was consistent (±3%) over the study years. Moreover, the lack of significant trends over time for some diseases examined, the decreasing trends for some illnesses, and the increasing trends for others suggest that these trends reflect real (albeit small) changes in the patterns and relative frequency of returned-traveler visits to specialist centers for these illnesses. In particular, the significant increase in proportion of ill travelers returning with enteric fever or dengue or seeking rabies PEP suggests that these conditions might have rising relevance for clinicians caring for ill returned travelers. For malaria, the average PM decreased significantly over the study period. Consistent regional trends were also seen, such as the high PMs for malaria among ill travelers returning from Oceania and sub-Saharan Africa, for enteric fever among those returning from south-central Asia, and for dengue among those returning from Southeast Asia (Figure 7). We also found significant clusters for malaria, enteric fever, and dengue.

Examination of simultaneous global changes in malaria epidemiology shows decreasing numbers of cases and deaths since 2000 (*10**,**11*). This decrease has been associated with increased funding for international malaria control, which has facilitated implementation of improved preventive and therapeutic interventions. Additionally, case numbers might have been overestimated, but they are now being rectified by increased use of rapid diagnostic tests (RDTs) and better case ascertainment.

One question is whether the observed overall decreasing trend in malaria PM is a travel medicine prevention success, a reflection of declining risk at destinations, a result of changes in diagnostics, or a combination of factors. Changes in diagnostic tests at GeoSentinel sites over the past decade, specifically more routine use of RDTs, are unlikely to have had much influence on malaria diagnosis at these specialized sites where experienced microscopists are available. Even if sensitivity of malaria diagnosis has increased with use of RDTs, this increase would not explain the observed decline in malaria PM. Because rates of imported malaria cases among travelers are influenced by use of personal protective measures and chemoprophylaxis, the decrease in PM might be partly attributable to better tolerated and more targeted prophylaxis (such as atovaquone/proguanil, available since 2000). However, the declining average PM trend over the study period (–28/1,000 ill travelers) is also consistent with reported global epidemiologic trends (*12*) resulting predominantly from decreased falciparum malaria cases. PMs for both falciparum and vivax malaria decreased significantly among ill persons returning after travel for tourism, visits to friends and relatives, and business.

Since 2009, resurgence of malaria cases has been observed in several African countries, including Rwanda, São Tomé and Príncipe, Zambia, and Cape Verde (*10*). Our data also showed a slight increase in malaria PMs over the past 2 years (Figure 3); this finding is consistent with European and US data on imported malaria PMs for 2008–2010 (*13**,**14*) but in juxtaposition to the reported global decline in case numbers.

About three quarters of all malaria cases occur in Africa (*10*); among cases reported here, 77% were acquired in sub-Saharan Africa. Case clustering was detected in 2000 and 2007 among travelers with malaria returning from Africa, but a review of electronic reports and relevant literature revealed no definitive specific corresponding outbreaks.

*S. enterica* ser. Typhi and Paratyphi cause an estimated 20 million cases of enteric fever and 200,000–600,000 deaths annually in disease-endemic countries; cases have been increasing globally (*15*). Growing drug resistance is compounding the associated public health problem (*16**,**17*). In industrialized countries, the proportion of travel-related cases has risen (*17**,**18*), particularly *S. enterica* ser. Paratyphi cases, which are not prevented by current typhoid fever vaccines. Our results showing that two thirds of cases were among ill travelers returning from south-central Asia and that enteric fever PM is increasing (+10/1,000, p = 0.013) are consistent with global trends (*19**,**20*).

In October 2009, cluster analysis for enteric fever detected greater importation than expected among ill travelers returning from Nepal. This increase was associated with a large outbreak among travelers from Israel who were vaccinated for *S. enterica* ser. Typhi (Vi vaccine) but who contracted S. enterica ser. ParatyphiA infection in a restaurant in Pokhara, Nepal (5).

Among GeoSentinel patients with dengue, 50% acquired infection in Southeast Asia; the PM for dengue (adjusted for the 2002 outbreak) increased significantly. Worldwide each year, 50–100 million dengue infections occur (*21*), nearly 75% in Southeast Asia and the Western Pacific region (*22*). In the past 50 years, reported incidence has increased 30-fold; dengue has expanded into new countries and into urban settings (*22**,**23*) associated with population growth, urbanization, development of periurban slums, movement of virus by infected travelers, and improved diagnostic capabilities (*21**,**24*). The marked increase in cases in dengue-endemic regions is also reflected by data reporting infection among international travelers; prospective seroconversion studies estimate attack rates among travelers to the tropics to be 1.0%–6.7% (*25**–**28*). However, improved awareness of dengue and improved diagnostics, especially with PCR and nonstructural protein 1 antigen testing now being routinely available, might well underpin the observed trend in dengue diagnoses among ill returned travelers. Travelers have also been reported to serve as sentinels for dengue infection: GeoSentinel data showed that travel-related dengue reflected defined regional seasonality, and natural annual oscillations of cases among populations in dengue-endemic regions were also observed among travelers to these regions (6). In 2002, an increase in travel-related dengue activity among GeoSentinel patients returning from Thailand was noted before an outbreak was recognized by official Thailand surveillance data (6). Increased dengue cases among ill returned travelers from south-central Asia in 2003 were also evident before official surveillance data were available. Not surprisingly, cluster analyses detected these cases, but our results additionally represent long-term trends in dengue reflected by traveler surveillance data.

Our data suggest an increase in PM for chikungunya. Interest in chikungunya fever, long known to be endemic to tropical Africa and Asia, resurged in 2005–2006 when a large outbreak spread through the Indian Ocean islands and Asia–Pacific region (*29**,**30*). The continuing epidemic has affected populations in popular travel destinations; many imported cases among travelers have been reported (*31*).

The significant rise in the PM of persons seeking rabies PEP, particularly ill travelers returning from Southeast Asia, might result from an increased absolute risk for animal bites or scratches, or from increased high-risk exposures, high-risk activities, or awareness of rabies risk among travelers resulting in more visits for PEP. Globally, the number of human rabies cases and deaths has decreased markedly over the past 20 years (*32**,**33*), but in parts of Indonesia (e.g., Bali) and China, it has increased (*34*).

Influenza clearly peaked in 2009, coinciding with the influenza (H1N1) pandemic. Although the underlying trend was not formally examined, the observed increase in number of cases (Figure 6, panel B) might reflect a real rise in the proportion of travelers acquiring influenza or might reflect a lowered threshold for referral to specialized clinics and better access to confirmatory diagnostics during this period.

This study has limitations. The GeoSentinel Surveillance Network captures data only on ill persons who visit specialized clinics, and these data do not represent all international travelers. GeoSentinel data can therefore not be used to calculate absolute risk; PM calculations are performed instead. PM is a complex measure and changes over time either because of changes in numbers of reported cases of the disease of interest or because of significant changes in other diagnoses. Patterns of travel also change over time and are influenced by political, economic, and cultural events. Consequently, interpretation of results is complex, and changes in PM reflect changes in the recognized levels of the specific illnesses seen at specialized sites over time rather than changes in absolute risk for disease acquisition. Where relevant, comments regarding changes in numbers of cases have also been included to verify that changes in frequency of other diagnoses do not explain reported PM trends. Because the relative case mix of patients and diagnoses differs by GeoSentinel site, analyses need to account for changes in visits to each site over time. Subanalyses were performed to ensure that no single site was unduly driving overall trends (data not shown). Because disease acquisition is affected by numbers of travelers to each destination, type and duration of travel, preventive measures implemented, and many other factors, traveler surveillance data would not be expected to precisely mirror trends in illness among host populations. 

Despite these limitations, the annual changes in PM, although small, showed statistically significant trends that correlate with regional trends in disease for many diagnoses examined. In particular, PM changes for 3 major travel-related illnesses reflect global trends in disease epidemiology; trends for malaria decreased and trends for enteric fever and dengue increased. When case numbers were sufficient, significant regional trends could also be detected. We have also shown that an algorithm for detecting case clusters can be used on longitudinal traveler surveillance data. These findings highlight how sentinel surveillance of travelers provides an additional layer in surveillance efforts that can be used to inform the international community about disease activity trends in disease-endemic areas. Additionally, the relative contribution of diagnoses among returned ill travelers from different regions provides useful information for provision of health advice before and after travel.
